# Pressure‐decay testing of pleural air leaks in intact murine lungs: evidence for peripheral airway regulation

**DOI:** 10.14814/phy2.13712

**Published:** 2018-05-24

**Authors:** Andrew B. Servais, Cristian D. Valenzuela, Alexandra B. Ysasi, Willi L. Wagner, Arne Kienzle, Stephen H. Loring, Akira Tsuda, Maximilian Ackermann, Steven J. Mentzer

**Affiliations:** ^1^ Laboratory of Adaptive and Regenerative Biology Brigham & Women's Hospital Harvard Medical School Boston Massachusetts; ^2^ Institute of Functional and Clinical Anatomy University Medical Center of the Johannes Gutenberg‐University Mainz Germany; ^3^ Department of Anesthesia, Critical Care, and Pain Medicine Beth Israel Deaconess Medical Center Harvard Medical School Boston Massachusetts; ^4^ Molecular and Integrative Physiological Sciences Harvard School of Public Health Boston Massachusetts

**Keywords:** Air leak, injury, lung, ventilation

## Abstract

The critical care management of pleural air leaks can be challenging in all patients, but particularly in patients on mechanical ventilation. To investigate the effect of central airway pressure and pleural pressure on pulmonary air leaks, we studied orotracheally intubated mice with pleural injuries. We used clinically relevant variables – namely, airway pressure and pleural pressure – to investigate flow through peripheral air leaks. The model studied the pleural injuries using a pressure‐decay maneuver. The pressure‐decay maneuver involved a 3 sec ramp to 30 cmH_2_0 followed by a 3 sec breath hold. After pleural injury, the pressure‐decay maneuver demonstrated a distinctive airway pressure time history. Peak inflation was followed by a rapid decrease to a lower plateau phase. The decay phase of the inflation maneuver was influenced by the injury area. The rate of pressure decline with multiple injuries (28 ± 8 cmH_2_0/sec) was significantly greater than a single injury (12 ± 3 cmH_2_O/sec) (*P* < 0.05). In contrast, the plateau phase pressure was independent of injury surface area, but dependent upon transpulmonary pressure. The mean plateau transpulmonary pressure was 18 ± 0.7 cm H_2_O. Finally, analysis of the inflation ramp demonstrated that nearly all volume loss occurred at the end of inflation (*P* < 0.001). We conclude that the air flow through peripheral lung injuries was greatest at increased lung volumes and limited by peripheral airway closure. In addition to suggesting an intrinsic mechanism for limiting flow through peripheral air leaks, these findings suggest the utility of positive end‐expiratory pressure and negative pleural pressure to maintain lung volumes in patients with pleural injuries.

## Introduction

Pleural air leak leading to pneumothorax is an important clinical issue for neonates, children, and adults. The incidence of pneumothorax in infants on positive pressure ventilation ranges from 22% to 41% with subsequent mortality rates as high as 31–70% (Sly and Drew 1984; Yu et al. 1986; Klingenberg et al. 2017). In both spontaneously breathing and mechanically ventilated patients, air leaks are a potentially life‐threatening complication in patients with cystic fibrosis (CF) (Flume et al. 2010), idiopathic pulmonary fibrosis (IPF) (Iwasawa et al. 2010), and lymphangioleiomyomatosis (LAM) (Almoosa et al. 2006). Air leaks are also the most common complication in all patients undergoing pulmonary surgery (Malapert et al. 2010; Petrella et al. 2016; Pompili and Miserocchi 2016).

The management of pleural air leaks in patients on mechanical ventilation is particularly challenging (Mentzer 2010). After control of the air leak with a tube thoracostomy (Mentzer et al. 2018), a common practice is to minimize pleural suction to limit air flow through the pleural fistula (Cerfolio et al. 2002). This approach, however, risks inadequate evacuation of the pneumothorax and compromised ventilation (Mentzer et al. 2018). Another approach is to increase pleural suction to ensure the optimal inflation of the lung. This approach, however, may delay healing of the pleural fistula as well as lead to the development of empyema (Liang et al. 2013; Gilbert et al. 2016).

Previous work defining the pathophysiology of peripheral air leaks has been limited. Fontan and Ray (1989) studied the pressure‐flow behavior of a peripheral bronchopleural fistula in an anesthetized lamb model. These investigators noted that fistula resistance was influenced by both lung volume and the pressure drop across the fistula. Walsh and Carlo studied air flow through a peripheral fistula in an anesthetized rabbit model. They found that transpulmonary pressure was an important determinant of fistula air flow, but could not define a predictable relationship between transpulmonary pressure and fistula air flow (Walsh and Carlo 1989). Ellsbury et al. studied high frequency ventilation in a neonatal piglet model. These investigators found that higher frequencies, short inspiratory times, and low mean airway pressures were associated with improved ventilation (Ellsbury et al. 2002).

In this report, we used a novel pressure‐decay leak testing method to explore the pathophysiology of pleural air leaks. The pressure‐decay testing was used to investigate the relevant impact of airway and pleural pressures on flow through peripheral air leaks. The data were analyzed in the context of transpulmonary pressures and regional airway closure.

## Methods

### Animals

Male mice, 8–10 week‐old wild‐type C57BL/6 (Jackson Laboratory, Bar Harbor, ME) were anesthetized prior to euthanasia (Gibney et al. 2011). The care of the animals was consistent with guidelines of the American Association for Accreditation of Laboratory Animal Care (Bethesda, MD) and approved by the Brigham and Women's Hospital Institutional Animal Care and Use Committee.

### Anesthesia and intubation

The animals were anesthetized with an intraperitoneal injection of Ketamine 100 mg/kg (Fort Dodge Animal Health, Fort Dodge, Iowa) and Xylazine 10 mg/kg (Phoenix Scientific, Inc., St. Joseph, MO). Prior to mechanical ventilation, the glottis was directly visualized and intubated with a standard 18 gauge Angiocatheter to minimize tracheal air leak (BD Insyte, Sandy, Utah). After intubation, the animal was transferred to a FlexiVent rodent ventilator (Scireq, Montreal, QC Canada) as previously described (Gibney et al. 2011).

### Pleural injury

The standard pleural injury was created with a 25 g needle (Becton Dickinson), with a diameter of 0.51 mm, inserted 1–2 mm into the mouse lung. The pleural injury was created in the cephalad division of the left lung. In some experiments, additional punctures were made in the same region to increase the total surface area of the injury. The surface area of the 25 g needle was 0.2 mm^2^ (equivalent to a 2.1 cm^2^ injury scaled to the average human lung surface)(Lindstedt and Schaeffer 2002).

### Pulmonary mechanics

Prior to all measurements, the pressure transducers and ventilator tubing of the FlexiVent (SciReq, Montreal, QC) were calibrated as described (Gibney et al. 2012). After general anesthesia and intubation, the mice were transferred to the FlexiVent system for pulmonary mechanics studies. The animals were hyperventilated at a rate of 200/min and allowed to acclimate to the ventilator for 2 min prior to standardization of the volume history with three consecutive positive pressure recruitment maneuvers. After recruitment, pulmonary mechanics studies were performed. Pressure‐volume loops were obtained starting from a PEEP of 3 cmH_2_O by an 8‐sec steady inflation ramp to 30 cmH_2_O with a 8‐sec passive deflation. Mechanical ventilation was briefly stopped and the animal passively exhaled to functional residual capacity.

### Pressure decay leak test

Similar to commercial pressure decay leak testing (Extrand et al. 2008; Shi et al. 2014), the lung was inflated to a target pressure (30 cmH_2_0), then isolated from the supply pressure. In the standard “inflation maneuver”, a 6 sec inflation maneuver was performed consisting of a 3 sec ramp to 30 cmH_2_O followed by a 3 sec detection phase. The monitored pressure drop reflected the air leak. The leak rate was calculated based on the best fit of the linear portion of the decay curve.

### Negative pressure chamber

The negative pressure chamber was a custom 15 × 15 × 5 cm acrylic chamber with vacuum control and water manometer pressure relief system (Atrium, Hudson, NH). Negative pressure range was 0 to −40 cmH_2_O and typically applied in 10 cmH_2_O increments.

### Statistical analysis

Data analysis was performed using XLstat (Addinsoft, New York, NY) add‐in for Microsoft Excel. Results were reported as mean ± one standard deviation unless otherwise noted. Pairwise comparisons were performed as needed, using Tukey's test. The significance level for the sample distribution was defined as *P* < 0.05.

## Results

### Pleural injury and air leak

**Figure 1:**
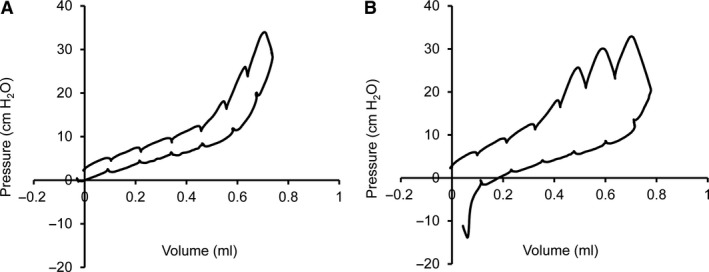
Pressure‐volume loops in a mouse before and after pleural injury with air leak. (A) A standard pressure‐volume loop was performed by the Flexivent ventilator provided an assessment of the mechanical properties of the lung. (B) After injury with a 25 g needle, the pressure‐volume loop is distorted by the air leak.

**Figure 2:**
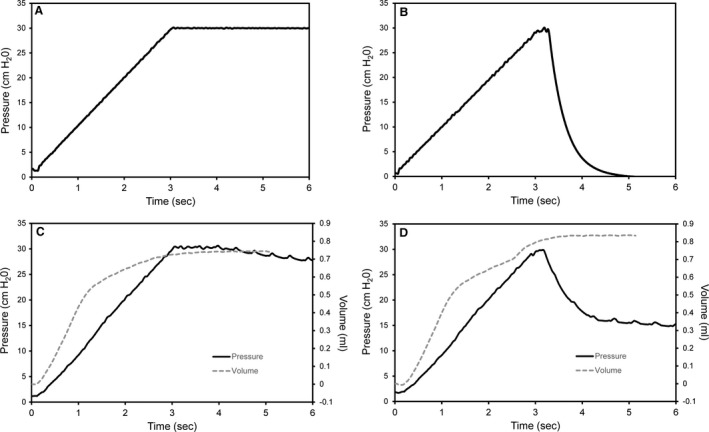
Standard inflation maneuver in simulacrum and in vivo. A baseline maneuver was performed followed by an identical maneuver with air leak. (A) An intact polyvinylidene chloride balloon was used to demonstrate the inflation maneuver to 30 cmH_2_0 followed by a standard pressure plateau. (B) The same polyvinylidene balloon with an air leak demonstrates exponential decay to 0 cmH_2_0. (C) In vivo, the baseline tracheal airway pressures (solid line) demonstrated a similar ramp and plateau. The cumulative volume delivered by the ventilator is shown (gray dashed line). The modest plateau pressure decline reflected the expected stress‐relaxation; cardiac movement is also more obvious during the pressure plateau. (D) After pleural injury with a 25 g needle, the ramp pressures approached the 30 cmH_2_0 peak pressure, but rapidly declined to a plateau pressure. The cumulative volume in the pleural injury is higher than in the uninjured tracing. Tracings from representative mice are shown.

In orotracheally intubated mice on mechanical ventilation, pleural injuries were produced by a 25 g needle inserted 1–2 mm into the lung parenchymal. Pressure‐volume (PV) curves were obtained both before and after the pleural injury (Fig. 1). As expected, the pleural injury resulted in a distorted PV curve with increased area between the inspiratory and expiratory phases of the loop (Fig. 1B). To simplify subsequent analyses, the pleural injuries were studied using a standard inflation maneuver. A polyvinylidene chloride balloon simulacrum illustrated the maneuver; specifically, a 3 sec ramp to 30 cmH_2_0 followed by a 3 sec breath hold. In the absence of an air leak, the breath hold was associated with a 3 sec pressure plateau at 30 cmH_2_0 (Fig. 2A). With an air leak, the balloon reached a peak pressure of 30 cmH_2_0, but subsequently demonstrated an exponential decline in airway pressure (Fig. 2B). In the mechanically ventilated mouse lung, the inflation maneuver resulted in the 3 sec ramp to 30 cmH_2_0 followed by a 3 sec plateau (Fig. 2C). After pleural injury, the inflation maneuver resulted in a distinctive airway pressure (*P*_aw_) time history; that is, peak inflation was followed by a rapid decrease to a lower plateau phase at 15–18 cmH_2_0 (Fig. 2D).

### Decay phase

**Figure 3:**
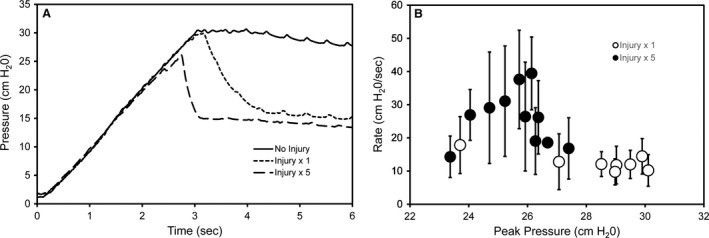
Pressure decline of standard inflation maneuver after single and multiple pleural injuries. (A) Tracheal pressures during an inflation maneuver in baseline condition (solid line), after a single pleural injury (dotted line), and after 5 injuries (dashed line). Representative tracings are shown. In both injury conditions, a decline in pressures (1) is followed by a plateau phase (2). (B) The rate of decay (decay rate = (peak pressure−plateau pressure)/time) plotted as a function of the peak pressure. Single pleural injuries (open circles) are compared to 5 injuries (closed circles); each point represents one mouse with error bars reflecting the mean of three estimates of the rate of decay ± 1 SD.

To determine the influence of injury surface area on *P*_aw_, single and multiple 25 g needle insertions were compared. As the injury surface area increased, the peak pressures achievable with the rodent ventilator decreased: no injuries 30.1 ± 0.1 cmH_2_0, 1 injury 29.2 ± 0.6 cmH_2_0, and 5 injuries 25.8 ± 1.1 cmH_2_0. In addition to peak pressures, the shape of the decay phase of the inflation maneuver appeared to be influenced by the injury area (Fig. 3A). The rate of pressure decline with multiple injuries (28 ± 8 cmH_2_O/sec) was significantly greater than a single injury (12 ± 3 cmH_2_O/sec) (*P* < 0.05; Fig. 3B). These data indicated that the decay phase of pressure‐decay testing reflected the size of the injury area.

### Plateau phase

**Figure 4:**
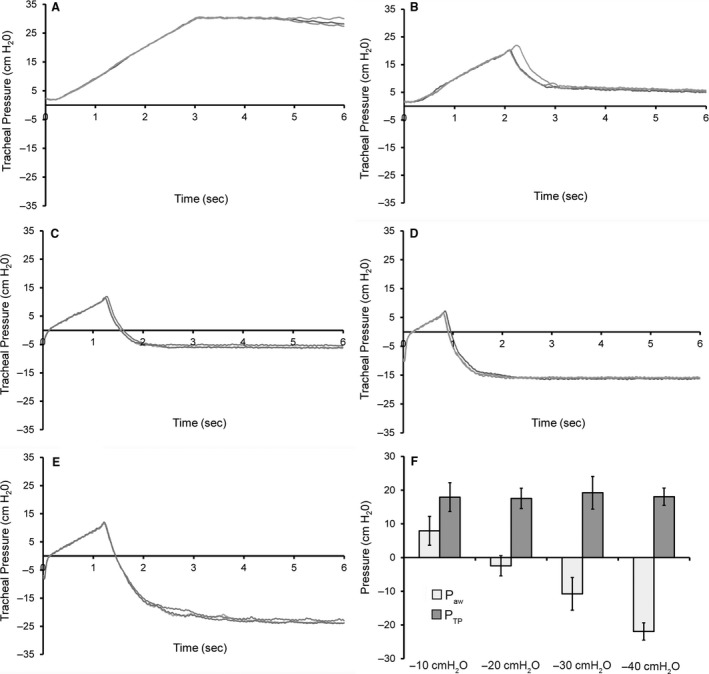
Transpulmonary pressure plateau after single pleural injury. Tracheal pressure plateau of standard inflation maneuver in mice with variable “pleural” pressures; that is, body box pressures outside of the lung after chest wall removal. (A) Inflation maneuver in a mouse with no pleural injury and a pleural pressure of 0 cmH_2_0. Plateau pressures are indistinguishable from baseline tracings. (B) A single pleural injury and a pleural pressure of −10 cmH_2_0 demonstrated decreased peak and plateau pressures. Similarly, pleural pressures of −20 cmH_2_0 (C), −30 cmH_2_0 (D), and −40 cmH_2_0 (E) demonstrated a progressive decrease in peak pressures and the plateau phase. Representative tracings of *N* = 3 mice are overlaid in each condition. (F) A summary of plateau transpulmonary pressure (*P*_TP__,_ dark gray), defined as tracheal pressure minus body box pressure, compared to plateau tracheal pressure (*P*_aw_, light gray). There was no significant difference between mean *P*_TP_ in the 4 conditions (*P* > 0.05). In contrast, mean transpulmonary pressures were significantly different from *P*_aw_ in each condition (*P* < 0.001). *N* = 3 mice; error bars reflect mean ± 1 SD.

To investigate the plateau phase of the pressure‐decay curves, we studied *P*_aw_ as a function of the pressure drop across the whole lung (transpulmonary pressure). To vary pressure on the outside of the lung, the chest wall was removed and the mice were placed in a pressure‐regulated body box – a negative pressure environment analogous to a chest tube on suction. The standard inflation maneuver was performed before and after pleural injuries and at a range of clinically relevant pleural pressures (0, −10, −20, −30 and −40 cm H_2_O). The peak airway pressures achieved by the ventilator were reduced by lower pleural pressures (Fig. 4A–E). Plateau pressures were also reduced by lower pleural pressures; however, the relationship between tracheal pressure and pleural pressure was relatively constant. The plateau transpulmonary pressures were remarkably similar (mean 18.1 ± 0.7 cmH_2_0) regardless of the absolute pressures outside of the lung (*P* < 0.001; Fig. 4F). The transpulmonary pressure regulation of the air leak flow indicated that leak volumes in the decay phase were limited by intrinsic properties of the lung.

### Inflation phase

**Figure 5:**
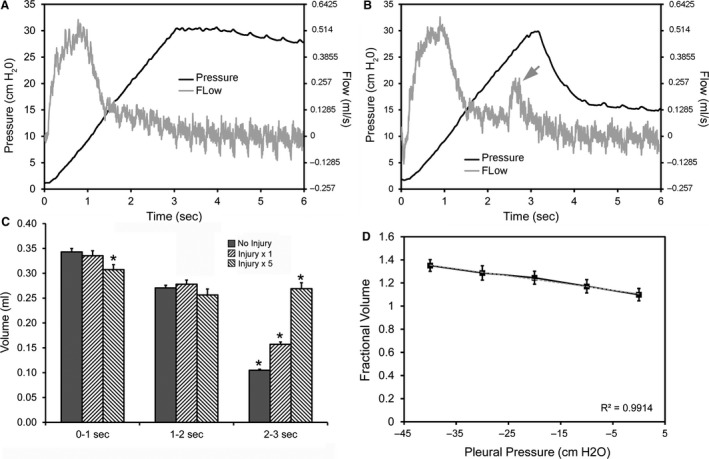
Air leak volume associated with single and multiple pleural injuries. (A) Baseline inflation maneuver (no injury) demonstrating the ventilator‐delivered air flow (gray line) and corresponding tracheal pressures (black line). As expected, the delivered air volume preceded the rise in tracheal pressures. (B) Representative inflation maneuver after a pleural injury (single) demonstrated a second peak of air delivery near the tracheal pressure peak (arrow). (C) The total air volume delivered by the ventilator during the inflation maneuver in three conditions was summarized in three time intervals (*N* = 4 mice). In the initial 1 sec of the inflation maneuver, less volume was delivered in the multiple injury condition, possibly related to compliance changes after injury (asterisk, *P* < 0.05). The major difference was the increased volume delivered in both the single and multiple injury (injury × 5) conditions during the 3rd second of the maneuver – the difference between all three conditions was highly significant during this interval (asterisks, *P* < 0.001). (D) When negative pressure outside the lung was applied during the inflation maneuver, the fractional air volumes delivered by the ventilator relative to baseline (no leak) controls increased linearly with decreasing pressure (*N* = 4 mice; *P* < 0.01 for each condition). Linear trendline is shown (gray dashes) (*R* ^2^ = 0.9914). Error bars reflect mean ± 1 SD.

In addition to conventional pressure‐decay testing, the FlexiVent permitted the analysis of delivered air flow during the 3 sec ramp of the inflation maneuver (Fig. 5A). In the air leak conditions, the initial delivered volume was similar to the no injury baseline condition; however, additional volume was delivered late in the inflation maneuver near peak inflation pressures (Fig. 5B, arrow). To quantify this observation, the delivered volumes were calculated for baseline conditions, single injury and multiple injuries during the 3 sec inflation maneuver (Fig. 5C). During the initial 2 sec of lung inflation, the delivered volumes were similar in the three conditions. Possibly reflecting injury‐related changes in lung compliance, the initial delivered volume in the multiple injury condition was slightly lower than baseline or single injury conditions (*P* < 0.05; Fig. 5C). During the last second of the inflation ramp (2–3 sec), the delivered volume was substantially greater in the multiple injury and single injury lungs than in the no injury control (*P* < 0.001, Fig. 5C). The addition of negative pleural pressure further increased the leak volume (Fig. 5D). A linear increase in leak volume was demonstrated between 0 and −40 cmH_2_O of pleural pressure (*P* < 0.001) (Fig. 5D). The maximum of −40 cmH_2_O of suction increased inflation leak volume 34 ± 3%.

## Discussion

Air flow through a pleural air leak has been commonly considered an interaction of pleural hole size, local lung compliance, airway pressure, relative airway resistance and transpulmonary pressure (Roth et al. 1988) – a complex interaction likely to preclude a practical understanding of its pathophysiology. Here, we adapted a pressure‐decay leak testing method to demonstrate three features of the pleural air leak. (1) The size of the pleural defect influenced the peak pressures and the rate of pressure decay. (2) The plateau phase of the pressure‐decay curve was largely independent of the size injury, but dependent on transpulmonary pressure. (3) The inflation, or pressurization, phase of the pressure‐decay testing demonstrated that nearly all leak volume was lost near peak inflation. We conclude that the air leak flow limitation at lower lung volumes reflects transpulmonary pressure‐dependent airway closure. These observations have practical implications for the clinical management of pleural air leaks.

There are several potential anatomic mechanisms contributing to flow limitation through a peripheral air leak. One possibility is that the pleural injury effectively eliminated the local contribution to the driving pressure for gas flow. Alveolar pressure, composed of the recoil pressure of the lung and pleural pressure, is the driving pressure that keeps airways open. When local recoil pressure is lost due to injury, the driving pressure is diminished and regional airway closure occurs. The recruitment of these peripheral airways requires higher lung volumes (end‐inflation); conversely, airway closure occurs at comparable transpulmonary pressures. A second and mutually compatible possibility is that the peripheral lung injury disrupts structural elements responsible for airway‐parenchymal interdependence (Pare and Mitzner 2012; Wagner et al. 2015). The local disruption of these elements would contribute to the impaired opening and early closure of airways subtending the injury.

In patients with a pleural air leak on mechanical ventilation, the pressure‐decay testing described here provides a practical method for assessing the physiologic characteristics of the air leak as well as a practical approach to ventilator management. During lung inflation, the majority of the leak volume is lost near peak inflation. This observation is consistent with previous studies (Walsh and Carlo 1989) and empirical observations (Ellsbury et al. 2002) of the utility of moderating peak inflation pressures to minimize air leaks. These observations also indicate that the use of end‐expiratory pressure will have little impact on leak volume. Although conventional practice is to minimize the negative pressure applied to a pleural chest tube (Cerfolio et al. 2002), our data suggests that negative pleural pressures have a graded impact on leak volume during inflation – an impact that must be weighed against the risk of an undrained pneumothorax. During lung exhalation, the adverse effect of negative pleural pressures appears to be limited by airway closure at modest transpulmonary pressures (~18 cmH_2_0). Collectively, these observations suggest that minimizing peak inflation pressures and controlling the pleural space with negative pleural suction is a rational approach to the management of pleural air leaks.

Air leak analysis by pressure‐decay is a common method in nonbiologic product testing (ASNT, 1997; Hart 2016). The pressure‐decay method pressurizes the test system until it reaches a set pressure, and then monitors the pressure decay (Extrand et al. 2008). In nonbiologic testing, the pressure‐decay method is a sensitive and reproducible measure of system leaks (Vinogradov et al. 2016). Our pressure‐decay method, involving a 3 sec ramp to 30 cmH_2_0 followed by a 3 sec pause (or “breath hold”), was similarly sensitive and reproducible. We anticipate that the pressure‐decay method will be useful in not only evaluating air leaks in clinical settings, but also testing the effectiveness of potential clinical interventions such as the application of pleural sealants.

Although the pressure‐decay testing method is novel, our findings are generally compatible with previous work. The regulation of air leak flow by transpulmonary pressure is consistent with the observations of Fontan and Ray (1989) in a lamb model and Walsh and Carlo (1989) in a rabbit model. In addition, our findings of increased volume loss at peak inflation is consistent within common clinical observations (Herschel et al. 1978; Primhak 1983; Pierson et al. 1986) and studies of alternative ventilation techniques (Ellsbury et al. 2002). An interesting inconsistency is Fontan and Ray (1989) findings that fistula resistance increases with lung volume – a finding that implies volume loss should be less at peak inflation. In the Fontan and Ray model, the fistula was surgically created by connecting a bronchiole to the pleural space. As noted by others (Ellsbury et al. 2002), this model may not represent the pathophysiology of peripheral air leaks and the dynamics of distal airways and alveoli; nonetheless, we consider this point an open question that deserves additional study.

The investigation of peripheral air leaks in mice has several practical limitations that caution over‐interpretation in humans. First, small peripheral air leaks, analogous to air leaks observed in most clinical settings, are too small to directly quantify air flow through the injury. Instead, we used the delivered air volumes from the computer‐controlled piston ventilator (FlexiVent) to infer the air volume lost through the peripheral injury. We assume that the parenchymal pressurization in mice is analogous to the effect of mechanical ventilation in humans; however, the dynamics of patient ventilation requires special consideration. Second, generalizing our findings of air leak flow limitation presumes comparable lung and pleural pressures in humans and mice. Although there are multiple species differences in airway structure and ventilation (Gibney et al. 2011), we suspect that the effects of a loss of alveolar pressure observed in mice will be similar in humans. Finally, the exposure of the entire lung to a negative pressure chamber is only remotely analogous to a pleural tube with applied suction in humans. Our observations may be best viewed as an idealized case of pleural suction.

Finally, our findings suggest a useful distinction between peripheral pulmonary air leaks and bronchopleural fistulas. Historically, the phrase “bronchopleural fistula” was used interchangeably with “bronchial stump fistula” (Stemmermann et al. 1951) – indicating an air leak from “the main bronchus” (Monod et al. 1951). The bronchial leak could be the result of failed surgical technique (Lilienthal 1918) or the consequence of bronchial erosion by tumor or infection (Adams et al. 1951; Murphy et al. 1952). Positive pressure ventilation in patients with substantial bronchial leaks often results in catastrophic ventilatory failure (Baumann and Sahn 1990; Pforr et al. 2016). In contrast, the air leak described here refers to a defect in the pleura, often associated with underlying lung disease, mechanical ventilation or surgical resection. The data suggest that patients with peripheral air leaks can be managed on mechanical ventilation using approaches designed to minimize air leaks and optimize lung volumes. Our observations indicate that the air leaks in the peripheral lungs provide a unique opportunity to incorporate regional airway regulation into the rational clinical management of airway pressures and pleural suction.

## Conflict of Interest

The authors have no conflict of interest.
